# Sex-Dependent Effects of 7,8-Dihydroxyflavone on Metabolic Health Are Associated with Alterations in the Host Gut Microbiome

**DOI:** 10.3390/nu13020637

**Published:** 2021-02-16

**Authors:** Priyanka Sharma, Guojun Wu, Deeptha Kumaraswamy, Natalie Burchat, Hong Ye, Yongjia Gong, Liping Zhao, Yan Y. Lam, Harini Sampath

**Affiliations:** 1 Rutgers Center for Lipid Research, New Jersey Institute for Food, Nutrition, and Health, New Brunswick, NJ 08901, USA; priyanka.sharma@rutgers.edu (P.S.); dk866@scarletmail.rutgers.edu (D.K.); nab165@gsbs.rutgers.edu (N.B.); hy296@rutgers.edu (H.Y.); 2 Center for Microbiome, Nutrition, and Health, New Jersey Institute for Food, Nutrition, and Health, New Brunswick, NJ 08901, USA; gary.guojun.wu@rutgers.edu (G.W.); yongjia.gong@rutgers.edu (Y.G.); liping.zhao@rutgers.edu (L.Z.); 3 Department of Biochemistry and Microbiology, Rutgers University, New Brunswick, NJ 08901, USA; 4 Department of Nutritional Sciences, Rutgers University, New Brunswick, NJ 08901, USA

**Keywords:** metabolic syndrome, intestinal microbiome, dysbiosis, polyphenols, sex differences

## Abstract

7,8-Dihydroxyflavone (DHF) is a naturally occurring flavonoid that has been reported to protect against a variety of pathologies. Chronic administration of DHF prevents high-fat diet (HFD)-induced obesity in female, but not male, mice. However, the mechanisms underlying this sexual dimorphism have not been elucidated. We have discovered that oral DHF supplementation significantly attenuates fat mass, hepatic lipid accumulation, and adipose tissue inflammation in female mice. In contrast, male mice were not protected from adiposity, and had a paradoxical worsening of hepatic lipid accumulation and adipose tissue inflammation upon DHF supplementation. Consistent with these sexually dimorphic effects on body weight and metabolic health, 7,8-DHF induced early and stable remodeling of the female intestinal microbiome. DHF supplementation significantly increased gut microbial diversity, and suppressed potentially detrimental bacteria, particularly *Desulfovibrionaceae*, which are pro-inflammatory and positively associated with obesity and inflammation. Changes in the female gut microbiome preceded alterations in body weights, and *in silico* analyses indicated that these early microbial changes were highly predictive of subsequent weight gain in female mice. While some alterations in the intestinal microbiome were also observed in male DHF-supplemented mice, these changes were distinct from those in females and, importantly, were not predictive of subsequent body weight changes in male animals. The temporality of microbial changes preceding alterations in body weight in female mice suggests a role for the gut microbiome in mediating the sexually dimorphic effects of DHF on body weight. Given the significant clinical interest in this flavonoid across a wide range of pathologies, further elucidation of these sexually dimorphic effects will aid the development of effective clinical therapies.

## 1. Introduction

One of the greatest threats to public health is the increasing prevalence of adult and pediatric obesity, which increases the risk of cardiometabolic diseases such as type 2 diabetes, hepatic steatosis, and atherosclerosis. In the context of this urgent public health challenge, there is growing interest in evaluating natural compounds that show promise for weight management and maintenance of metabolic health [1,2]. One such naturally occurring compound is the flavonoid 7,8-dihydroxyflavone (DHF), which is enriched in several plants, including *Godmania*, *Tridax*, and *Primula* species [3,4,5]. DHF is a mimetic of brain-derived neurotrophic factor (BDNF) [3,4,5,6,7,8,9], which confers beneficial effects centrally through the tropomyosin-related kinase receptor B (TrkB) signaling pathway. DHF has demonstrated promising results as an antidepressant [10,11], in recovery from traumatic brain injury [12], and for the prevention of age-related cognitive decline and neurodegenerative disease [13,14,15,16,17]. Reports on the actions of DHF outside the brain are scarce, despite the known expression of TrKB receptors in the liver, neuromuscular junction, pancreas, small intestine, adipose tissue, and colon [9,18,19,20].

A recent study reported a novel sexually dimorphic role for DHF in body weight attenuation.Female mice fed a high-fat diet (HFD) along with oral DHF gained significantly less weight than vehicle-treated females; this same obesity resistance was not observed in male mice [21]. However, the mechanisms underlying this apparent sexual dimorphism in metabolic protection are not fully understood.

We asked the question of whether the gut microbiota may play a role in mediating the differential metabolic effects of DHF in male and female mice. Orally-administered DHF, like many other flavonoids, is poorly absorbed. Reported measures of absorption indicate that about 4% of ingested DHF is absorbed in mice [4], and thus its long transit time in the colon makes it likely to impact the gut microbial community [22,23,24]. Importantly, the gut microbiota may be particularly relevant to sex-specific effects—sex is known to be a key determinant of gut microbiota composition [25,26], and there is emerging evidence on the role of gut microbiota in sexual dimorphism, e.g., in modulating immunity [27,28] and cardiometabolic functions [29]. In this study, we hypothesized that DHF may exert differential effects on the gut microbiota in male and female mice, and sought to explore its relationship with metabolic phenotypes in the context of HFD-induced obesity.

## 2. Materials and Methods

### 2.1. Animal Studies

Age-matched male and female C57Bl6/J mice were used throughout the studies. 13-week old micewere given *ad libitum* access to an HFD (Research Diets D12451; 45% kcals fat, 20% kcals protein, 35% kcals carbohydrate; 5.8% fiber; 4.7 kcal/g metabolizable energy) and drinking water containing vehicle or 1 mg/mL DHF for 12-weeks. This translates to an approximate dosage of 650 mg/day for a 60 kg human [30], and represents a supraphysiological or pharmacological dose, guided by the low bioavailability of DHF and prior studies regarding its central effects that reported a similar dose [13,31]. 7,8-DHF was purchased from Tokyo Chemical Industry (TCI America, Portland, OR, USA), and was prepared as previously described [13,31]. Briefly, 7,8-DHF was dissolved in 17% dimethylsulfoxide (DMSO, ThermoFisher Scientific, Waltham, MA, USA) in phosphate-buffered saline (PBS) to generate a stock solution of 100 mg/mL; stocks were kept frozen. To prepare drinking water solutions, 1 mL stock solution was diluted in 100 mL water containing 1% sucrose (pH 8.0) and stirred overnight. Control animals received drinking water containing 1% sucrose and 0.17% DMSO/PBS. The HFD was replaced weekly, and the drinking solutions were freshly prepared and replaced biweekly. Two animals were lost during the study due to dermatitis and malocclusion, which was not attributable to the treatment. The final cohort sizes were as follows: control male *n* = 5, DHF male *n* = 4, control female *n* = 4, and DHF female *n* = 6.

Body weights and food intake were assessed weekly, and water intake was assessed biweekly. Body composition was determined by NMR (Echo MRI, Columbus, OH, USA), as previously described [32]. For in vivo procedures, all efforts were made to minimize discomfort and suffering, in accordance with approved animal care protocols. The breeding and care of the animals were in accordance with the protocols approved by the Animal Care and Use Committee of Rutgers University, New Brunswick, New Jersey. Following 12 weeks of feeding, mice were euthanized by isoflurane overdose, followed by cervical dislocation. Tissues were rapidly collected, fixed in 10% formalin or snap-frozen in liquid nitrogen, and stored at −80 °C until further analysis.

### 2.2. Lipid and Adipokine Analyses

Plasma triglycerides (TG) were measured using a colorimetric assay (ThermoFisher Scientific) after 12 weeks of HFD-feeding. Hepatic lipids were extracted by a modified Folch method and separated by thin-layer chromatography (TLC), as previously described [33,34]. Briefly, 20 mg liver tissue was homogenized in chloroform:methanol (2:1), followed by phase separation by the addition of acidified saline and centrifugation. The lipid fraction was dried, reconstituted in chloroform:methanol (2:1), spotted onto Silica Gel 60 chromatography plates, and developed in heptane:isopropyl ether:acetic acid (60:40:3) until the solvent front reached 1 cm from the top of the plate. The plates were dried, dipped in 10% copper sulfate in 10% phosphoric acid, air dried, and charred at 110 °C for 30 min. For gas chromatographic analyses, the TLC plates were visualized under UV light after spraying with dichlorofluorescein; bands corresponding to free fatty acids (FFA), cholesterol esters (CE), TG, and diacylglycerols (DAG) were scraped. Lipids were transmethylated in the presence of 14% boron trifluoride, and analyzed by gas chromatography-mass spectrometry (GC-MS) in order to assess their fatty acid content and composition, as previously described [33,34], using an Agilent 7870A gas chromatograph coupled with an Agilent 5977 mass spectrometer (Agilent Technologies Santa Clara, CA, USA). Plasma leptin (RayBiotech, Peachtree Corners, GA, USA) and adiponectin (Abcam, Cambridge, UK) were measured by ELISA.

### 2.3. Histology and Immunohistochemistry (IHC)

Fresh liver and adipose tissue slices were fixed in 10% neutral-buffered formalin for 24 h, followed by paraffin embedding. Five µm sections were stained with hematoxylin and eosin (HE) to visualize tissue architecture and lipid droplets, as previously described [35]. Immunohistochemistry for F4/80 was performed using the avidin–biotin complex method. Tissue sections were deparaffinized, boiled in citrate buffer for antigen retrieval, and incubated with a primary antibody against F4/80 (Abcam, Cambridge, MA, USA), followed by biotinylated anti-rat IgG secondary antibody (Vector Biolabs, Burlingame, CA, USA). F4/80 was detected using 3,3’Diaminobenzidine (DAB) as a substrate, counterstained with hematoxylin, and imaged by brightfield microscopy. Negative controls were incubated without primary antibody.

### 2.4. Gut Microbiota Analyses

Fresh fecal pellets were collected after 4 and 12 weeks of HFD and DHF/vehicle feeding, snap-frozen in liquid nitrogen, and stored at −80°C until analysis. Genomic DNA was extracted using the QIAmp Power Fecal DNA kit (QIAGEN, Germantown, MD), as per manufacturer’s instructions. The hypervariable region V4 of the 16S rRNA gene was amplified using the 515F and 806R primers modified by Parada et al. [36] and Apprill et al. [37] respectively, and was sequenced using the Ion GeneStudio S5 (ThermoFisher Scientific). Pprimers were trimmed from the raw reads using Cutadapt [38] in QIIME 2 [39]. Amplicon sequence variants (ASVs) [40] were obtained by denoising using the dada2 denoise-single command in QIIME 2, with the parameters --p-trim-left 0 -–p-trunc-len 215. Spurious ASVs were further removed by abundance filtering [41]. A phylogenetic tree of ASVs was built using the QIIME 2 commands alignment mafft, alignment mask, phylogeny fastree, and phylogeny midpoint–root in order to generate Weighted UniFrac metrics. Taxonomy assignment was performed using the q2-feature-classifier plugin [42] in QIIME 2, based on the silva database (release 132) [43]. The data were rarified to 24,000 reads/sample for subsequent analyses.

Overall gut microbiota structure was evaluated using alpha diversity indices (Shannon index, observed ASVs, and Faith’s phylogenetic diversity) and a beta diversity distance metric (weighted UniFrac). Principal coordinates analysis (PCoA) was performed using the R ‘ape’ package [44] to visualize differences in gut microbiota structure between the treatment groups along the principal coordinates that accounted for most of the variations. Random Forest analysis was performed and cross-validated using the R ‘randomForest’ package [45] and the ‘rfcv’ function, respectively, to test for correlations between gut microbiota composition and body weight. Key ASVs that contributed to variations in body weight were identified by redundancy analysis (RDA) using the R ‘vegan’ package [46]. The figures were visualized using the R ‘ggplot2′ [47] and ‘pheatmap’ packages [48].

Co-abundance networks were constructed to evaluate interactions between the gut bacteria within the ecosystem. In each group, ASVs shared by >30% of the samples were considered prevalent, and were selected for analysis. Pairwise correlations among the ASVs were calculated using the method described by Bland and Altman [49]. Correlations with *p* < 0.01 were included in the network analysis and visualized using Cytoscape v3.7.1. Eigenvector centrality was calculated by CytoNCA [50]. Kolmogorov-Smirnov tests were used to test for difference between the cumulative distribution curves. PageRank algorithm was applied to rank the nodes and identify the hubs [51].

### 2.5. Statistical Analyses

Mouse phenotype data are expressed as mean ± SEM for biological replicates; statistical comparisons were carried out using two-way ANOVA, followed by post-hoc analysis (Bonferroni)in Graph Pad Prism (version 8.2.0 for Windows, GraphPad Software, La Jolla, CA, USA). All statistical analyses of gut microbiota were performed in R. Specifically, alpha diversity at different time points within the same treatment group was compared using the Wilcoxon matched-pairs signed-ranks test (two-tailed), and alpha diversity of either the same treatment or sex groups at the same time point was compared using the Mann-Whitney test (two-tailed). Comparisons of the gut microbial community dissimilarity were performed using permutational multivariate analysis of variance (PERMANOVA). *p*-values < 0.05 were considered significant.

### 2.6. Data Availability

All data generated or analyzed during this study are included in this published article and its associated Supplementary Materials files. The raw gut microbiome sequencing data were deposited to the sequence read archive at NCBI under the BioProject ID PRJNA657894.

## 3. Results

### 3.1. Oral DHF Attenuates Weight Gain and Adiposity in HFD-Fed Female Mice, but Exacerbates Lipid Accumulation and Adipose Inflammation in Male HFD-Fed Animals

**Figure 1:**
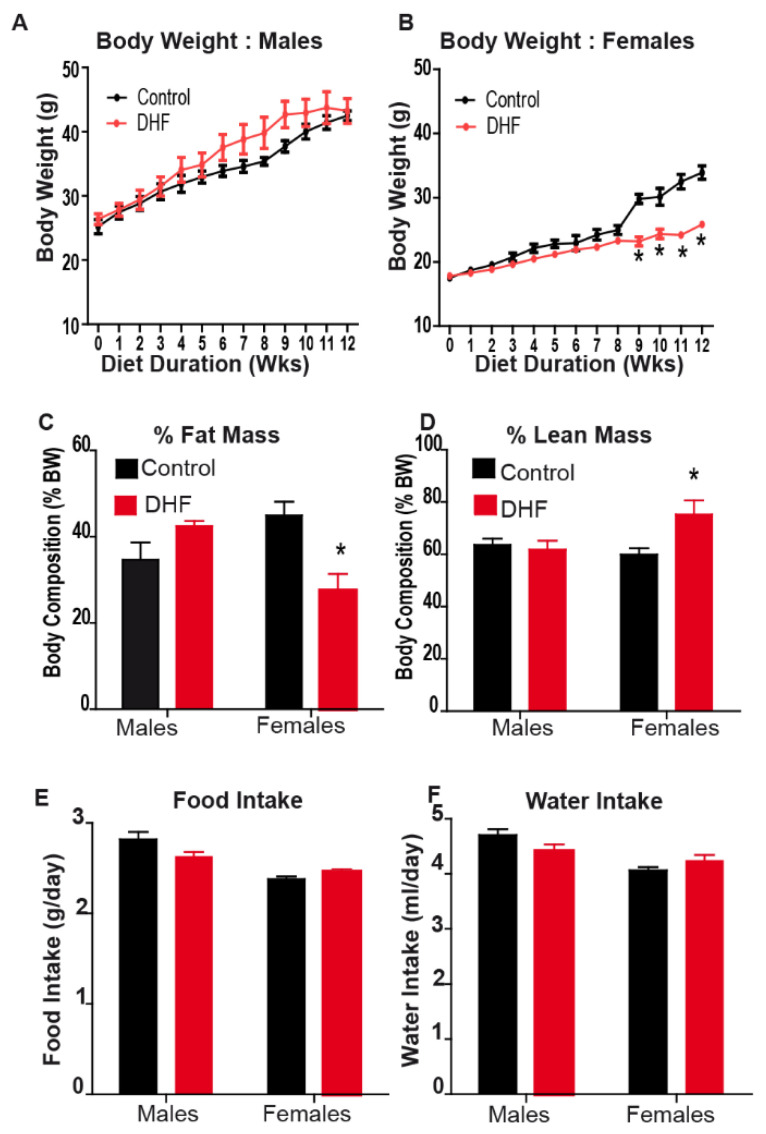
Oral 7,8-DHF confers protection against diet-induced obesity in female mice. Male and female mice were maintained on a high-fat diet (HFD) for twelve weeks, with or without oral 7,8-DHF (1 mg/mL). (**A**,**B**) Body weights were measured weekly, and (**C**,**D**) body composition was determined by NMR at the end of 12 weeks. (**E**) Food intake was measured weekly, and (**F**) water intake was measured biweekly during the feeding period. Data are expressed as average ± SEM (*n* = 4–6). Data were analyzed by ANOVA, followed by Bonferroni post-hoc comparison; * *p* < 0.05 vs. sex-matched controls.

Male and female mice were fed an obesogenic high-fat diet (HFD) with or without oral DHF. DHF was administered at a dose of 1 mg/mL in drinking water for 12 weeks, consistent with doses used in prior studies of cognitive function [13,31]. Consistent with a prior report [21], we observed that oral DHF administration attenuated diet-induced weight gain in female, but not male, mice (Figure 1A,B). This was further accompanied by a significant reduction in fat mass and preservation of lean body mass in female DHF-fed mice;male DHF-fed mice were not similarly protected (Figure 1C,D). No differences in food or water intake were observed between control and DHF-treated mice of either sex (Figure 1E,F). We also observed that female HFD-fed control mice had a significant increase in their rate of weight gain between weeks 8 and 9. A similar trend was reported by Chan et. al. [21], and we observed similar changes in the rate of weight gain at 8-week time point in other female HFD-fed cohorts (Sharma and Sampath, unpublished). Mechanisms underlying this inflection point in weight gain in female mice are not clear, but may be related to alterations in insulin sensitivity, potential alterations in intestinal microbial communities, and/or critical changes in metabolic rates at these time points during HFD-feeding in females. Regardless, DHF-fed females did not demonstrate this increased trajectory of weight gain, and were largely protected from HFD-induced weight gain, relative to control females (Figure 1B).

**Figure 2:**
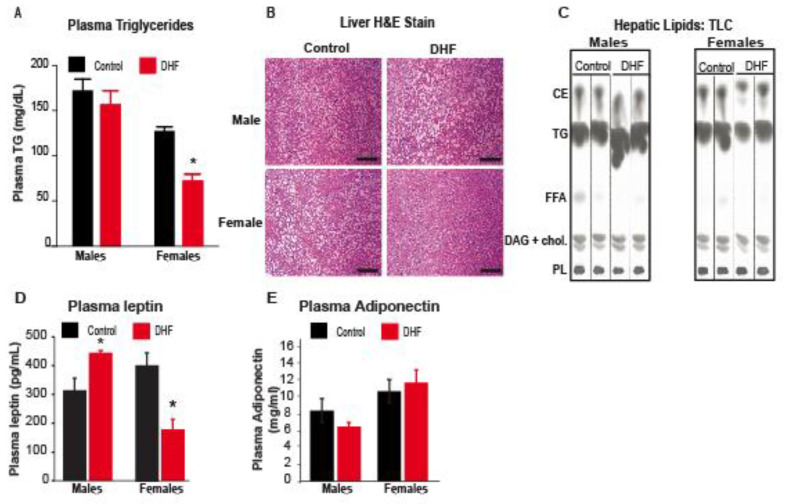
Plasma and hepatic lipids and plasma leptin are reduced in DHF-fed female mice. (**A**) Plasma triglyceride (TG) was measured colorimetrically. Data are expressed as average ± SEM (*n* = 4–6). Data were analyzed by ANOVA, followed by Bonferroni post-hoc comparison; * *p* < 0.05 vs. sex-matched controls. (**B**) Formalin-fixed liver sections were paraffin-embedded, cut into 5 µm sections, and stained with H&E; scale bar represents 100 µm. Images are representative of 4–6 mice per cohort. (**C**) Hepatic lipids were separated by thin layer chromatography (TLC). CE, cholesterol esters; DAG, diacylglycerols; FFA, free fatty acids; PL, phospholipids; TG, triglycerides. (**D**,**E**) Plasma leptin and adiponectin were measured by ELISA.

Consistent with the obesity resistance in female mice, DHF-fed females also had reduced plasma TG compared to vehicle-treated controls (Figure 2A). In addition, hepatic lipid droplets were smaller and less numerous in DHF-fed females, relative to control females (Figure 2B). Further analyses of hepatic lipids by TLC (Figure 2C) and GC-MS confirmed a significant reduction in total TG and free fatty acids (FFA) in DHF-fed females (Table 1), relative to control animals. The 43% reduction in total liver TG (Table 1) was primarily associated with a significant decrease in 16-carbon and 18-carbon lipids (not shown). The 24% reduction in liver FFA was driven by significant decreases in 14-, 16-, and 18-carbon saturated fatty acids (Table 1). DAG (Table 1) and CE (not shown) were not significantly altered by DHF in female mice. Consistent with the lack of protection from HFD-induced weight gain and adiposity (Figure 1A,C), DHF feeding did not alter serum TG or hepatic lipids in DHF-fed males (Table 1, Figure 2). Paradoxically, hepatic lipid accumulation was significantly increased in DHF-fed male mice, relative to vehicle-treated controls (Figure 2B,C). Hepatic TG content was increased by over two-fold in DHF-treated males (Table 1), primarily due to increases in 14–18 carbon lipid species (not shown). Interestingly, hepatic content of DAG was also increased by 2.5-fold in DHF-treated males, relative to control males (Table 1). As in the female mice, CE was not significantly altered in male animals (not shown). Plasma leptin levels were significantly reduced in female DHF-supplemented animals, while male DHF-treated mice had an increase in plasma leptin levels (Figure 2D). At this time point, plasma adiponectin levels were not significantly different between groups (Figure 2E).

**Figure 3:**
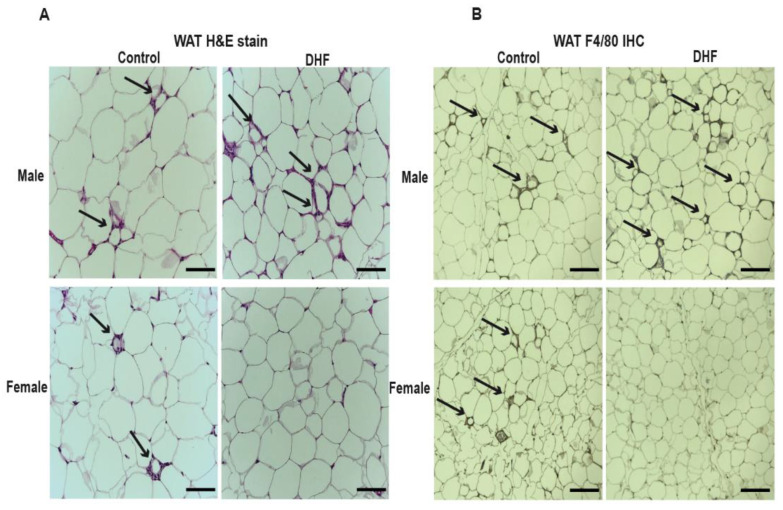
Adipose inflammation is reduced in 7,8-DHF-fed female mice. (**A**) Formalin-fixed sections of white adipose tissue (WAT) were paraffin-embedded, cut into 5 µm sections, and stained with HE for the visualization of their crown-like structures (black arrows), and (**B**) with F4/80 antibody to stain their macrophages (black arrows). The scale bar represents 100 µm. The images are representative of 4–6 mice per cohort.

Apart from reductions in hepatic lipid accumulation and leptin levels, DHF-fed females had a markedly reduced appearance of crown-like structures in their gonadal adipose depots (Figure 3A), suggesting decreased adipose inflammation. These crown-like structures were further confirmed to be pro-inflammatory loci through staining for the F4/80 macrophage marker. F4/80 staining, which is associated with macrophage infiltration into adipose tissue, was significantly attenuated in DHF-fed females (Figure 3B). Crown-like structures and F4/80 staining appeared to be elevated in adipose of DHF-fed males (Figure 3A,B). This is the first report of a potentially detrimental effect of DHF on male metabolic health, in the context of a HFD, as the previous studies did not investigate these parameters in male mice [21,52].

### 3.2. DHF Induces Early and Significant Remodeling of the Female Intestinal Microbiome

**Figure 4:**
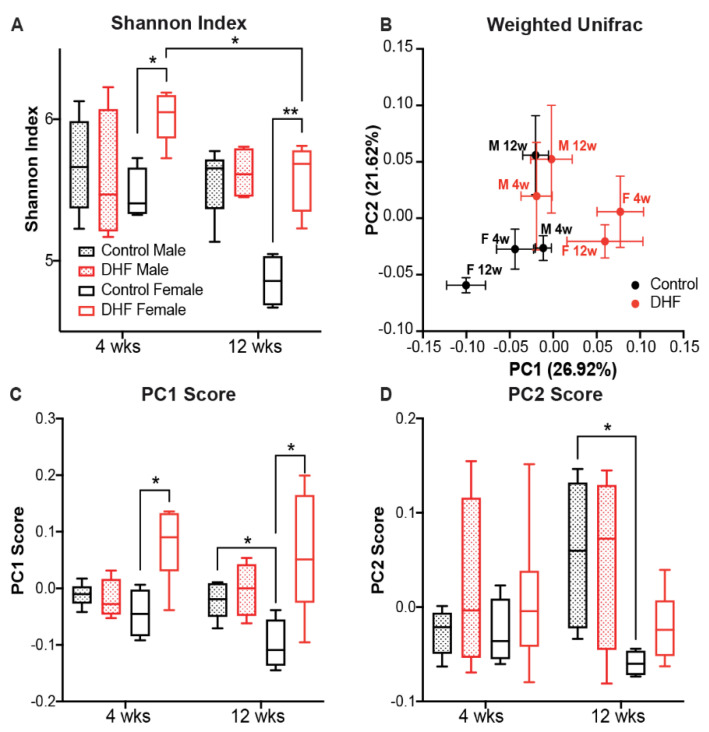
Gut microbiota composition is altered by 7,8-DHF in a sexually dimorphic manner. Overall gut microbiota structure was evaluated using (**A**) the Shannon index and (**B**) principal coordinates analysis based on the Weighted UniFrac distance. In panel B, each data point represents the mean principal coordinate (PC) score, and the error bar represents the SEM. (**C**,**D**) The scores of PC1 and PC2 in the principal coordinates analysis. The boxes show the medians and the interquartile ranges (IQRs); the whiskers denote the lowest and highest values that were within 1.5 times the IQR from the first and third quartiles. Data at different time points within the same treatment group were compared using the Wilcoxon matched-pairs signed-ranks test (two-tailed), and that of either the same treatment or sex groups at the same time point were compared using the Mann-Whitney test (two-tailed). * *p* < 0.05; *n* = 4–6/group.

We investigated changes in the gut microbiome upon DHF treatment by sequencing the 16S rRNA gene V4 region. A total of 38 mouse fecal samples from all four groups were sequenced. After denoising and abundance-based filtering, 634 reliable ASVs were retained for further analysis. DHF prominently changed the gut microbiota in female mice (Figure 4 and Supplementary Figure S1). Compared to sex-matched controls, DHF significantly increased the gut microbial diversity in female mice at week 4, as evidenced by a higher Shannon index (*p* < 0.05) and phylogenetic diversity (*p* < 0.05). Such differences between the control and treatment groups were maintained at week 12. In male mice, DHF had no impact on gut microbial diversity at week 4 (Figure 4A and Supplementary Figure S1). Observed ASVs and phylogenetic diversity in DHF-treated males were significantly higher than those in sex-matched controls at week 12 (*p* < 0.05; Figure S1). However, the Shannon index, which considers both membership and abundance, was similar at both time points (Figure 4A). These data suggest that the bacterial strains modulated by DHF in male mice were low in abundance, and therefore had minimal impact on the overall gut microbiota composition. In addition, we found a significant reduction in microbial diversity in DHF-treated female mice from week 4 to week 12 (*p* < 0.05 for both Shannon index and observed ASVs). This is likely a result of the obesogenic diet in female mice, as a similar trend was also observed in the sex-matched controls. No such difference in microbial diversity across the time points was observed in male mice (Figure 4A and Supplementary Figure S1A).

Beta diversity analysis was performed to assess the global effects of DHF on gut microbiota structure. Here, we focused on community dissimilarities based on the weighted UniFrac distance, which considers differences in membership, phylogenetic distances, and abundances. In the PCoA, PC1 and PC2 together accounted for 48.5% of the variations in the gut microbial community (Figure 4B). The gut microbiota in DHF-treated female mice and their controls showed significant separation at both week 4 (*p* = 0.013) and week 12 (*p* = 0.023), primarily along PC1 (Figure 4B–D). DHF did not induce further changes in the gut microbiota between the two time points (*p* = 0.335 for week 4 vs. week 12 in DHF-treated female mice). In contrast, there was no difference in the overall gut microbiota structure between the DHF-treated and control male mice at either time point (*p* = 0.176 for week 4 and *p* = 0.141 for week 12). These results show that DHF treatment induced significant and stable changes in the gut microbiota structure from 4 weeks onwards in female mice, but had no significant global effect in male mice.

### 3.3. The Bacterial Responders to DHF Supplementation Are Different between Female and Male Mice

**Figure 5:**
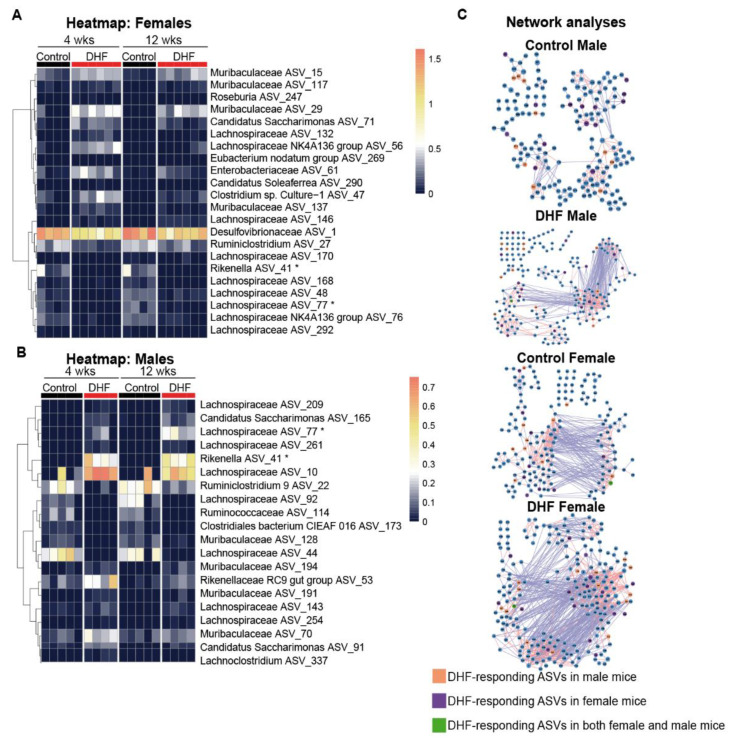
DHF-responding ASVs and microbial networks differ in male and female mice. The heatmaps show the log10-transformed relative abundance of the 22 7,8-DHF-responding ASVs in (**A**) female mice and (**B**) 20 ASVs in male mice from the redundancy analyses. (**C**) For each group, ASVs shared by more than 30% of the samples were included in the co-abundance networks. Pairwise correlations among the ASVs were calculated using the method described by Bland and Altman [53]. Correlations with *p* < 0.01 were included in the networks. The lines represent positive (red) and negative (blue) correlations between the nodes.

Specific bacteria that responded to DHF treatment were identified by redundancy analysis (RDA) (Supplementary Figure S2). Using DHF and time as the constraining variables, together they accounted for a similar percentage of variations in the gut microbiota in females (29.70%) and males (29.54%). Next, the ASVs which had at least 50% of their variability explained by these two constraining variables were identified, as they were likely to be the key contributors to the overall community changes. Here, 22 DHF-responding ASVs were identified in the female mice; among the 13 positive responders, four were from *Muribaculaceae*, four were from *Lachnospiraceae*, and the rest were from five other families; among the nine negative responders, six were from *Lachnospiraceae*, and the rest were from three other families (Figure 5A). In the male mice, 20 DHF-responding ASVs were identified (among the 14 positive responders, seven were from *Lachnospiraceae*, three were from *Muribaculaceae*, two were from *Rikenellaceae,* and two were from the phylum *Candidatus Saccharimonas*; among the six negative responders, two were from *Lachnospiraceae,* and the rest from four other families; Figure 5B). Most of these ASVs were identified as DHF-responders only in either the male or female mice, even though they were present in both sex groups. At week 4, 17 of the 22 DHF-responding ASVs in the females were found in the control male mice, and 18 of the 20 DHF-responding ASVs in the males were found in the control female mice. Only two ASVs were identified as DHF-responders in both sexes: *Rikenella* ASV_41 and *Lachnospiraceae* ASV_77. Interestingly, while these two ASVs were downregulated by DHF in the female mice, they were significantly upregulated by DHF in the male mice. Another notable differential effect of DHF in male and female mice was on ASV_1 in the family *Desulfovibrionaceae,* which produce endotoxin and hydrogen sulfide. ASV_1 was highly abundant in the female controls (21.0% in week 4 and 29.2% in week 12) and was significantly reduced at both time points in the DHF-treated females (8.9% in week 4 and 13.2% in week 12). This bacterium was also abundant in the male mice but was not affected by DHF (Control vs DHF, 18.8% vs. 16.5% in week 4, and 10.2% vs. 16.2% in week 12).

### 3.4. Both Sex and DHF Alter the Gut Microbiota Co-Abundance Network

As microbes do not exist in isolation, but rather form complex interaction networks [53], relationships between bacteria might play a role in how the gut microbiota respond to DHF. For each group, we constructed a co-abundance network of the prevalent ASVs, which were shared by >30% of the samples, in order to examine the ecological interactions of the gut microbiota [54]. There were more direct interactions among members of the gut microbiota in female mice. DHF treatment increased such interactions in both sex groups, but sex-dependent differences were retained. As shown in Figure 5C, the average number of neighbors in females was 5.4 and 3.2 in males, and the characteristic path length was 5.608 in females and 8.062 in males. This indicates that the microbial network in the female controls has significantly higher complexity and compaction compared to male counterparts. DHF further increased the complexity and compaction in both the male and female mice (Figure 5C; Supplementary Table S1). The average number of neighbors was increased by over 140%, and the characteristic path length was decreased by over 30% when the control and DHF-modulated networks were compared within each sex. The complexity and compaction remained higher in female than male mice after DHF treatment (Supplementary Table S1). Eigenvector centrality, a measure of the relative importance and connectivity of each node [55], also showed that more bacteria were connected with each other in the female than the male gut microbiota. DHF treatment further increased this connectivity in both sexes (Kolmogorov-Smirnov tests; *p* < 0.05; Supplementary Figure S3), but consistently more so in female mice.

Focusing on DHF-responding ASVs and their neighbors in the network, the same DHF-responding ASVs were found to have distinct neighbors and very different correlations with the other bacteria in each network (Supplementary Tables S2 and S3). For example, for *Desulfovibrionaceae* ASV_1, which negatively responded to DHF in female mice, its neighbors were changed from one *Lachnospiraceae* ASV_78 (positively correlated) in the control female mice to *Candidatus Saccharimonas* ASV_52, *Lachnospiraceae* ASV_152, and *Lactobacillus* ASV_341 (all negatively correlated) in the DHF-treated female mice. This indicates that the DHF treatment changed the relationship between ASV_1 and the other members of the gut microbiota. In the male mice in which *Desulfovibrionaceae* ASV_1 was not affected by DHF, its neighbors were *Lactobacillus* ASV_9 and *Muribaculaceae* ASV_176 in the DHF-treated group, and *Muribaculaceae* ASV_164 and *Ruminococcaceae* UCG-010 ASV_350 in the control group. Such differences in the local neighbors of the same ASV across the networks indicate differences in the ecological interactions among the bacteria, which might contribute to the differential responses to DHF between the male and female mice.

### 3.5. DHF-Responding Bacteria Predict Body Weight Changes in Female Mice

**Figure 6:**
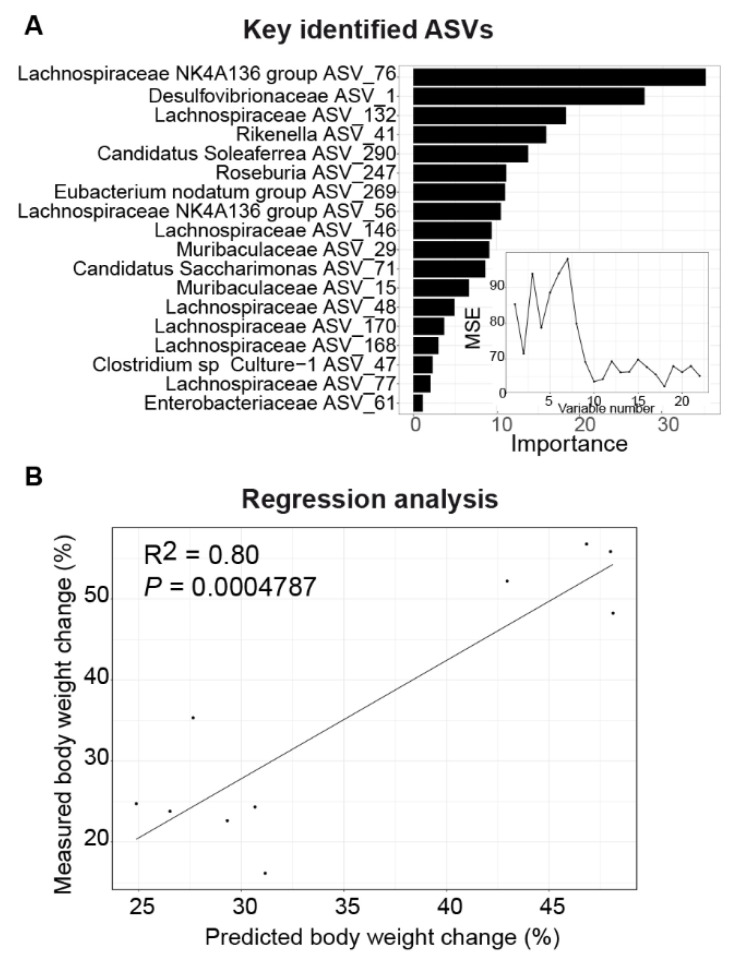
Gut microbiota alterations are highly predictive of body weight change in female mice. (**A**) Random Forest (RF) model regressing the percent body weight changes (from week 4 to week 12) on the abundance of the RDA-identified key ASVs (at week 4). The RF assigns a mean error rate, or a feature-importance score, to each function; this value indicates the extent to which each ASV contributes to the accuracy of the model. The subpanel shows the number of variables and the mean squared error of the corresponding model. (**B**) Scatter plot of the measured and predicted percent body weight changes.

Since the alterations in the gut microbiome were apparent at 4 weeks in the female mice (Figure 4), prior to any divergence in body weight (Figure 1B), we asked the question of whether these early changes would be predictive of the change in body weight following this time point. We used the week 4 abundance of the 22 key ASVs identified in the female gut microbiota to predict the changes in body weight from week 4 to week 12 using a Random Forest regression in the female mice. Based on cross-validation and feature selection, 18 ASVs were selected as predictors for the best model (Figure 6A). A significant correlation was observed between the measured and predicted percentage weight change (R^2^ = 0.80; *p* = 0.0004784; Figure 6B), supporting a role for the DHF-modulated gut microbiome in regulating body weight. To test whether this relationship between the gut microbiota and body weight regulation was unique to females, this regression was repeated using the DHF-responding ASVs and body weight changes in the male mice, but no significant correlation was found (R^2^ = 0.26; *p* = 0.1572). These data indicate that DHF induced stable and early modifications to the female microbiome that preceded the resistance to obesity observed in female mice. The temporality of these changes indicates a potentially causal role for the microbiome in conferring obesity resistance in DHF-fed females.

## 4. Discussion

We show here that oral DHF administration protects female, but not male, mice from HFD-induced obesity. Conversely, HFD-fed male mice were not only resistant to metabolic protection by DHF, they accumulated more hepatic lipids than control males. In particular, levels of hepatic TG—the primary storage form of hepatic lipids—were increased 2-fold in DHF-fed males, indicating that this flavonoid may exacerbate diet-induced fatty liver disease in male mice. In addition, hepatic content of DAG was increased 2.5-fold in male mice. Since increased hepatic DAG is directly associated with insulin resistance [56], this significant increase in hepatic DAG in male DHF-fed mice may contribute to impaired glucose homeostasis in these animals. Sex-dependent effects of DHF have been reported in a small number of prior publications. For instance, Chan et al. [21] reported that female mice were resistant to obesity upon DHF supplementation, as corroborated by our current study. Another study reported that DHF conferred greater neuroprotection following hypoxia in female mice, relative to their male counterparts [57]. However, while these studies only reported a lack of protective effects in male mice, our study indicates that DHF in fact exacerbates metabolic dysfunction in male mice, without significantly increasing body weight or fat mass. The current study is thus the first to demonstrate a potentially detrimental effect of DHF in male mice, in the context of high-fat feeding. Such differences were not observed in the prior reports regarding DHF and metabolic health, as male mice were largely omitted from these prior investigations [21,52]. However, our paradoxical observations of worsened metabolic health in male DHF-fed mice underscore the importance of studying both male and female mice in further elucidating sex-based differences in response to this clinically attractive flavonoid. In our current study, this apparent exacerbation of metabolic phenotypes was only determined in HFD-fed animals. Any potential detrimental effects of DHF in chow-fed or lean mice has not yet been investigated, but may be of clinical relevance.

We also show here for the first time that the metabolic protection observed in DHF-fed female mice was associated with significant remodeling of the gut microbiota structure. Furthermore, we observed significant reductions in potentially detrimental bacteria in the female, but not male, mice. Importantly, this remodeling occurred at early time points, prior to the divergence in body weight, and was highly predictive of subsequent weight gain in DHF-fed female mice. No such consistent or predictive remodeling of the gut microbiome was observed in male DHF-fed mice, consistent with their lack of metabolic protection. An emerging body of literature supports a causative role for gut microbiota in obesity and its deleterious metabolic sequelae [58,59,60,61,62]. In our dataset, we identified nine different ASVs from *Desulfovibrionaceae*, with *Desulfovibrionaceae* ASV_1 being the most predominant member, which accounted for ~99% of the total abundance of this family. DHF supplementation in female mice significantly reduced *Desulfovibrionaceae* ASV_1 by more than 50%, but was without effect in male mice. Members of *Desulfovibrionaceae*, which produce endotoxin and hydrogen sulfide and are pro-inflammatory, have been reported to be positively associated with obesity and inflammation [62,63,64]. In addition, *Desulfovibrionaceae* has previously been shown to be abundant in HFD-induced obese mice and was reduced by various flavonoid-containing compounds that were protective against obesity, such as seabuckthorn freeze-dried power [65] and aged citrus peel extract [66]. Another bacterium that differentially responded to DHF in female and male mice was *Rikenella* ASV_41, a member of a genus that has recently been shown to be positively correlated with obesity and inflammation markers in HFD-induced obese mice [67]. In the current study, *Rikenella* ASV_41 was significantly reduced in female, but increased in male, mice by DHF, and these changes were concomitant with the differential effects of DHF on hepatic lipid accumulation in females and males (Figure 2). Similar associations between *Rikenella* abundance and hepatic steatosis have been reported in other mouse interventional studies [62,68,69], suggesting that modulation of the gut microbiota—which then impacts hepatic lipid metabolism—may be a key mechanism by which DHF confers protection against the metabolic sequelae of high-fat feeding.

A key question raised by these findings is that of how DHF differentially modulates the gut microbiota in male and female mice. We showed that the majority of the DHF-responding gut bacteria identified in male or female mice were in fact present in both sexes. This suggests that, rather than being merely a consequence of membership differences, other sex-specific mechanisms determine the bacterial response to the same oral stimulus. From a microbial ecology perspective, gut bacteria interact with each other, and this intertwining network may impact the ways in which the gut bacteria are directly or indirectly modulated. In the current study, we applied co-abundance network analysis to explore the ecological interactions between microbes [54]: a positive correlation implies co-occurrence, which could be a mutualistic, commensalistic or parasitic relationship, whereas a negative correlation may suggest a co-exlusion relationship, which could be competitive or antagonistic. While the network *per se* does not inform the underlying mechanisms of the observed relationships, it is a tool for the exploration of bacteria–bacteria interactions, and for hypothesis generation. In the current study, the female gut microbiota had a more complex network compared to that of the male mice (Figure 5C). While DHF supplementation increased the density and size of the networks in both sexes, it further enlarged the difference between male and female gut microbiota. Overall, the gut microbiota network topology has been reported to be associated with host phenotypes. In a cohort study with 551 subjects, a higher number of network edges were associated with lower BMI [70]. It is thus plausible that the more complex gut microbiota interactions in female mice are at least partially responsible for the more favorable metabolic effects of this flavonoid in females. In addition to the difference in global topological features, we found that the same DHF-responding ASVs had largely distinct neighbors across networks in the different sex and treatment groups. This suggests that the same bacteria may have different niches in the gut microbial community in female and male mice, which may contribute to their differential responses upon DHF treatment. A notable example is *Desulfovibrionaceae* ASV_1, which was up-regulated by DHF in male mice, but down-regulated in female mice by DHF. *Desulfovibrionaceae* ASV_1 had only one positive neighbor (*Lachnospiraceae* ASV_78) in the control female mice, but it had three negative neighbors in the DHF-treated female mice (*Candidatus Saccharimonas* ASV_52, *Lachnospiraceae* ASV_152 and *Lactobacillus* ASV_341). Amongst these negative neighbors was one ASV from the *Lactobacillus* genera. Many members in *Lactobacillus* have been reported to be beneficial in the alleviation of HFD-induced obesity [71]. Compared with the control, *Lactobacillus* ASV_341 tended to be higher in the DHF-treated female mice. It is possible that DHF promote beneficial bacteria, which also suppress detrimental bacteria, and as a result, confer protection against HFD-induced obesity in female mice.

In our studies, the divergences in body weight curves were not apparent at the onset of the DHF feeding, but rather became evident after about 7–9 weeks of supplementation (Figure 1B). Similarly, the previous study by Chan et. al. [21] reported an almost 13-week delay in protection upon DHF administration, when a much lower (0.16 mg/mL) oral DHF dose was used. Interestingly, a recent study administering low-dose DHF to obese mice also reported a lag of 5–7 weeks before differences in body weight were apparent [52]. These consistent findings in multiple labs of a significant period of delay are intriguing. Given the short half-life of DHF [10], it is not likely that this lag period results in any significant serum or tissue accumulation of DHF. Rather, we hypothesize that this delayed protection from obesity is further supportive of a period of intestinal microbial remodeling by DHF which precedes, and may be required for, its effects on body weight. Further gnotobiotic studies, such as those involving reciprocal fecal material transplants between male and female DHF-treated mice, would clarify the extent to which the microbiome mediates the observed obesity-resistance phenotype in females, or indeed the lack of metabolic protection in male mice. In addition, a limitation of our current study was the somewhat limited sample size, even though all statistical analyses accounted for the numbers of mice in each cohort. Nevertheless, additional studies with larger numbers of both male and female mice may further clarify the role of the gut microbiome in either worsening or improving metabolic health in response to DHF supplementation. Lastly, DHF, like other flavonoids, is subject to further metabolism in the liver, small intestine, and colon [22,23]. 7,8-DHF predominantly undergoes glucuronidation and methylation in the liver and gut [10,14], with methylated derivatives such as 8-methoxyflavone and 7-methoxyflavone having reported half-lives that are comparable to the parent compound [10,14] and retaining the ability to activate TrKB receptors, [10]. Sex differences in the metabolism of DHF have not been previously reported, nor were they the focus of our current study. These studies are challenged by the short half-lives of both DHF and its metabolites [4] However, future studies investigating whether DHF is metabolized differentially by male vs. female hosts, and whether such differences correspond to metabolic phenotypes, are warranted.

## 5. Conclusions

In summary, our results are the first to demonstrate a potentially detrimental metabolic effect of the flavonoid 7,8-DHF in male HFD-fed mice. Our report is also the first demonstration of a sexually dimorphic effect of 7,8-DHF on the intestinal microbiome. Importantly, they point to a role for the early sex-dependent remodeling of the gut microbiome as a potential mechanism mediating the subsequent obesity resistance in female mice. Such differences in the mode of action are of particular relevance to the development of personalized nutrition strategies for the prevention and mitigation of metabolic diseases. Furthermore, given the known effects of both DHF [15,16,17] and the gut microbiota [72,73] on the central nervous system, these findings may have broader implications for the clinical use of this compound across other pathologies.

## Figures and Tables

**Table 1:** Hepatic lipid content.

	Males		Females	
µg/g	Control	HFD + DHF	Control	HFD + DHF
**TG**	45,855.19 ± 6077.03	95,189.23 ± 10,876.28 *	64,168.04 ± 4124.24	36,878.28 ± 11,150.35 *
**DAG**	1573.77 ± 490.91	3925.14 ± 1357.26 *	2543.88 ± 102.3	2276.79 ± 329.91
**FFA**	927.4 ± 100.9	1147.2 ± 251.33	1471.82 ± 106.83	1123.69 ± 54.1 *

Hepatic lipid content and composition by GC-MS. Hepatic triglyceride (TG), diacylglycerol (DAG), and free fatty acid (FFA) content were quantified by GC-MS. Data are expressed as average ± SEM (*n* = 4–6) and wereanalyzed by ANOVA, followed by Bonferroni post-hoc comparison; * *p* < 0.05 vs. sex-matched controls.

## Data Availability

All data generated or analyzed during this study are included in this published article and its associated Supplementary Materials files. The raw gut microbiome sequencing data were deposited to the sequence read archive at NCBI under the BioProject ID PRJNA657894. The data that support the findings of this study are available from the corresponding authors upon reasonable request.

## Supplementary Materials

The following are available online at https://www.mdpi.com/2072-6643/13/2/637/s1. Figure S1. Change of gut microbiota membership. (A) The number of observed amplicon sequence variants (ASVs). (*B*) Faith’s phylogenetic diversity. The boxes and whiskers are denoted as per Figure 5, and the outliers are shown as individual data points. The data at different time points within the same treatment group were compared using the Wilcoxon matched-pairs signed-ranks test (two-tailed), and those of either the same treatment or sex groups at the same time point were compared using the Mann-Whitney test (two-tailed). * *p* < 0.05 and ** *p* < 0.01. *n* = 4–6/group. Figure S2. Triplot of the redundancy analysis (RDA) of the microbiota composition in male (A) and female (B) mice. As the environmental variables, 7,8-DHF and time were entered. The samples are indicated by open triangles and circles. The amplicon sequence variants (ASVs) with at least 50% of the variability in their abundance explained by RDA1 and RDA2 are indicated by blue arrows. Figure S3. Cumulative distribution curves of the eigenvector centrality in each network. Kolmogorov–Smirnov tests were performed to test the differences. Table S1. Topological parameters of each network. Table S2. Neighbors of 7,8-DHF-responding ASVs in the female mice in different networks. Table S3. Neighbors of 7,8-DHF-responding ASVs in the male mice in different networks.
