# Supplementary material for: Sex-Dependent Effects of 7,8-Dihydroxyflavone on Metabolic Health Are Associated with Alterations in the Host Gut Microbiome
Source: Nutrients. 2021 Feb 16;13(2):637. doi: 10.3390/nu13020637 (PMC7920311; doi:10.3390/nu13020637)
Supplement: Supplementary file 1 [file nutrients-13-00637-s001.zip › Sharma et al._Supplementary Legends.pdf]

Supplementary figure S1. Change of gut microbiota membership. (*A*) The number of observed amplicon sequence variants (ASVs). (*B*) Faith's phylogenetic diversity. Boxes and whiskers were denoted as per Figure 5 and outliers are shown as individual data points. Data at different time points within the same treatment group were compared using the Wilcoxon matched-pairs signed-ranks test (two-tailed) and that of either the same treatment or sex groups at the same time point were compared using the Mann-Whitney test (two-tailed). * $p < 0.05$ and ** $p < 0.01$. *n*=4-6/group.

Supplementary figure S2. Triplot of redundancy analysis (RDA) of the microbiota composition in male (*A*) and female (*B*) mice. 7,8-DHF and time were entered as environmental variables. Samples are indicated by open triangles and circles. Amplicon sequence variants (ASVs) with at least 50% of the variability in their abundance explained by RDA1 and RDA2 are indicated by blue arrows.

Supplementary figure S3. Cumulative distribution curves of eigenvector centrality in each network. Kolmogorov-Smirnov tests were performed to test the differences.

Supplementary Table S1. Topological parameters of each network.

Supplementary Table S2. Neighbors of 7,8-DHF-responding ASVs in female mice in different networks

Supplementary Table S3. Neighbors of 7,8-DHF-responding ASVs in male mice in different networks
