# Supplementary figures and images for: Sex-Dependent Effects of 7,8-Dihydroxyflavone on Metabolic Health Are Associated with Alterations in the Host Gut Microbiome
Source: Nutrients. 2021 Feb 16;13(2):637. doi: 10.3390/nu13020637 (PMC7920311; doi:10.3390/nu13020637)

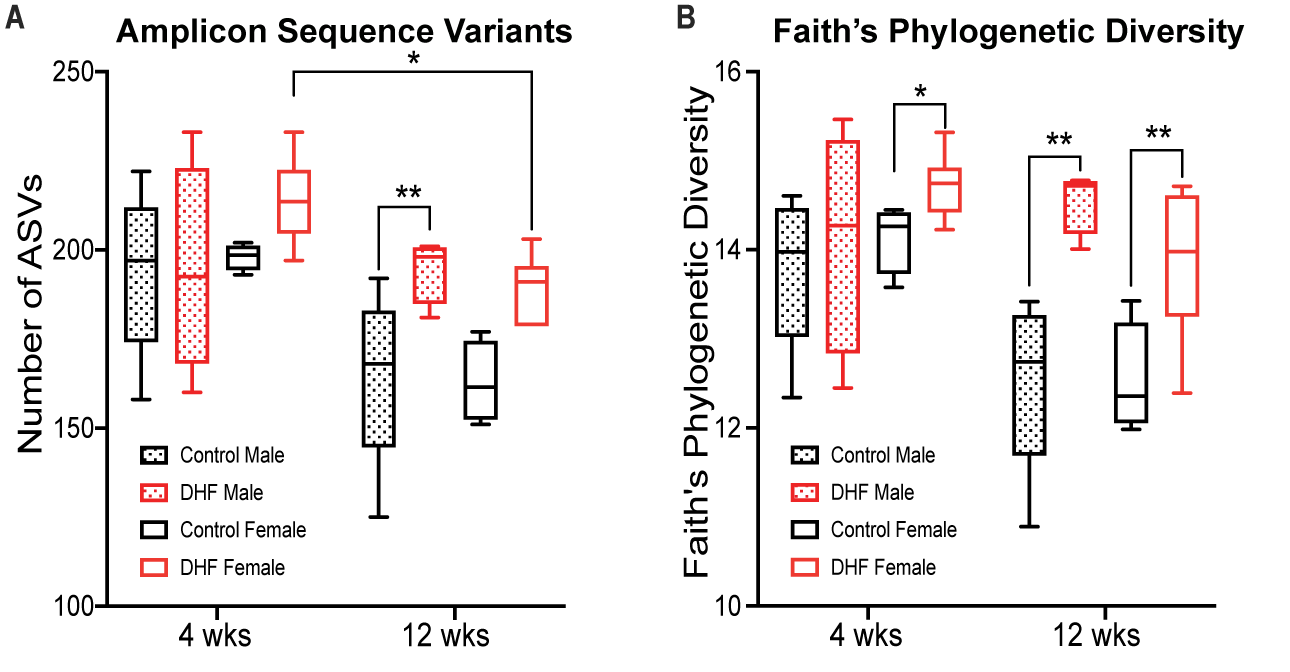

Supplement: Supplementary file 1 [file nutrients-13-00637-s001.zip › Figure S1_Double column_color.tif]

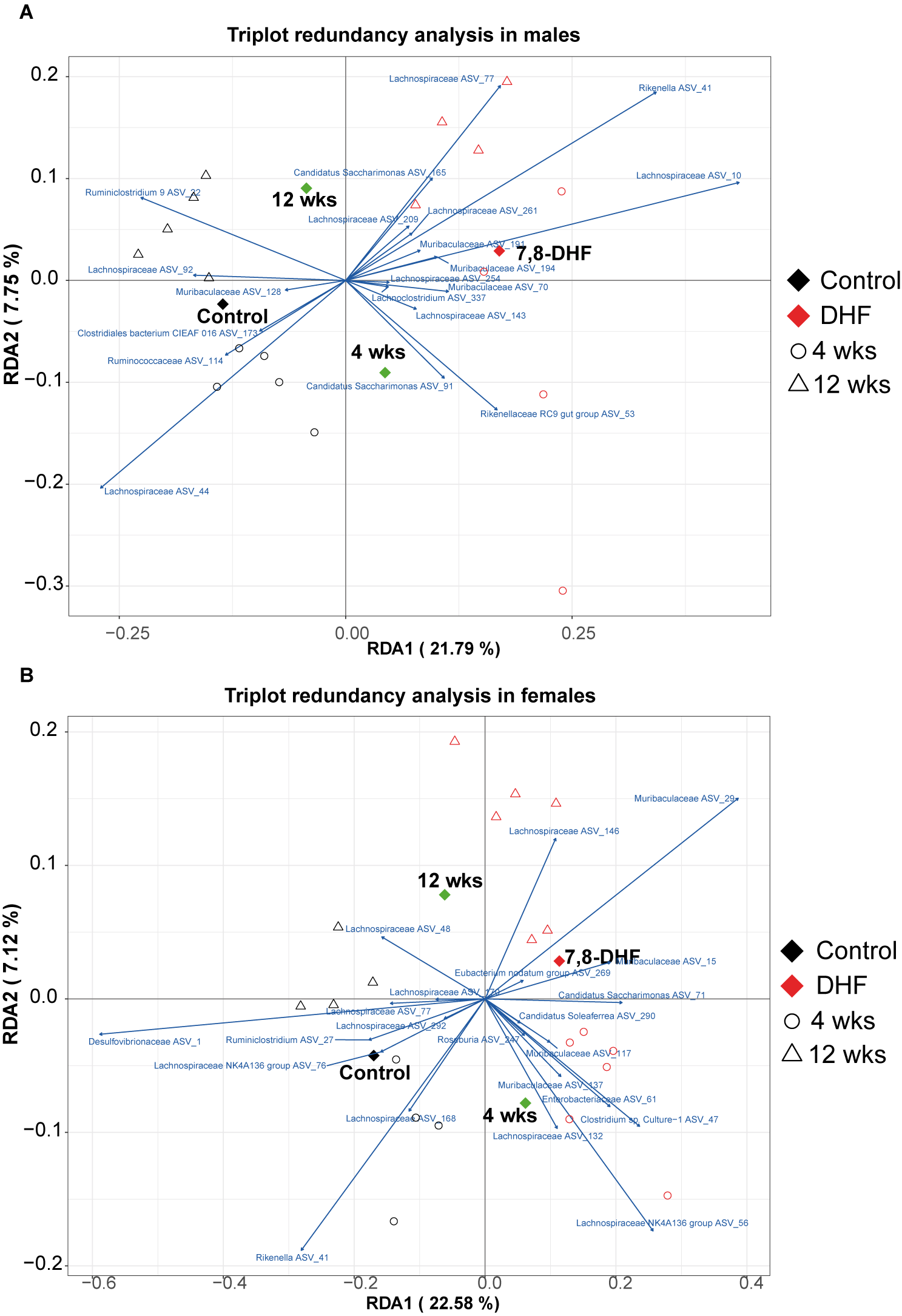

Supplement: Supplementary file 1 [file nutrients-13-00637-s001.zip › Figure S2_Double column_Gray.tif]

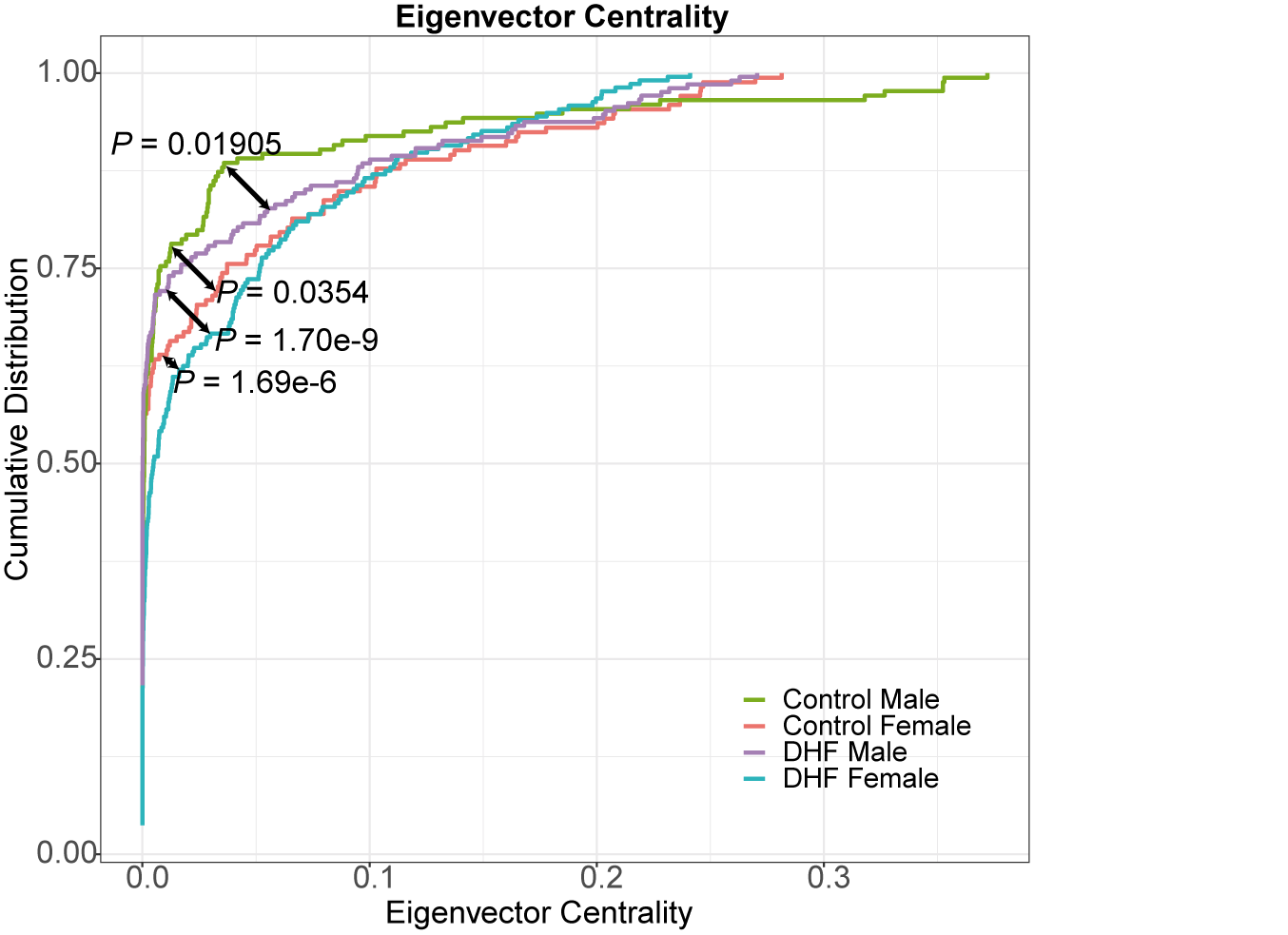

Supplement: Supplementary file 1 [file nutrients-13-00637-s001.zip › Figure S3_Single_color.tif]
